# Severity of frailty as a significant predictor of mortality for hemodialysis patients: a prospective study in China

**DOI:** 10.7150/ijms.51569

**Published:** 2021-07-25

**Authors:** Wenjing Fu, Aihua Zhang, Lina Ma, Linpei Jia, Jagadish K Chhetri, Piu Chan

**Affiliations:** 1 Department of Nephrology, Beijing Institute of Geriatrics, Xuanwu Hospital of Capital Medical University, Beijing, China; 2 Department of Neurobiology, Beijing Institute of Geriatrics, Xuanwu Hospital of Capital Medical University, Beijing, China; 3 Department of Geriatrics, Beijing Institute of Geriatrics, Xuanwu Hospital of Capital Medical University, Beijing, China; 4 National Clinical Research Center for Geriatric Disorders, Beijing, China

**Keywords:** Frail, Hemodialysis, Aging, Ultrafiltration volume, Mortality, Chinese

## Abstract

**Background:** Frailty is known to be highly prevalent in older hemodialysis (HD) patients. We studied the prevalence of frailty and its associated factors in Chinese HD patients. We further studied if frailty could predict survival in HD patients.

**Methods:** This is a prospective study involving patients receiving maintenance HD in the dialysis center of Xuanwu Hospital, Beijing. Study subjects were enrolled from October to December, 2017 and followed up for two years. Demographic data, comorbidities and biological parameters were collected. Frailty was assessed using the Fried frailty phenotype at baseline. Cox regression analysis was performed to identify the relationship between frailty and mortality in HD patients. Kaplan-Meier was plotted using the cutoff value obtained by ROC curve to evaluate survival rates in different frailty status.

**Results:** Total of 208 HD patients were enrolled with a mean age of 60.5±12.7 years. According to the frailty criteria, at baseline the prevalence of robust, pre-frail and frail in HD patients was 28.7%, 45.9%, and 25.4%, respectively. The two-year all-cause mortality was 18.8% (39/207) and underlying causes of death included coronary artery disease (CAD), cerebrovascular disease (CVD), hyperkalemia, severe infection, malignant tumor and others. Survival curve showed the patients with frailty score ≥4 to have significantly shorter survival time as compared to patients with frailty score ≤ 3. Frailty predicted two-year mortality when frailty score ≥4 with a sensitivity of 70% and a specificity of 83.67% with an AUC of 0.819. Frailty score was positively associated with age and ratio of ultrafiltration volume to dry weight, while negatively associated with levels of serum albumin, uric acid and diastolic blood pressure after HD.

**Conclusions:** Our results confirm frailty to be very common among HD patients and severity of frailty was a significant predictor of mortality for HD patients. Factors such as age, malnutrition and low blood pressure are the factors to be associated with frailty. Interdialytic weight gain inducing excessive ultrafiltration volume is an important risk factor.

## Introduction

Frailty is considered as a geriatric syndrome of multiple system dysregulation associated with aging 1, a physiological state of increased vulnerability to stressors 2, 3. Frailty is characterized by a loss of physiologic reserve and associated with increased risks of adverse outcomes such as falls, hospitalization, prolonged hospital stays and even mortality 4. Compared to robust persons, frail individuals may have difficulty to quickly recover from chronic diseases 1, 2, 4.

The prevalence of chronic kidney disease (CKD) is increasing worldwide due to population aging. Initiation of hemodialysis (HD) has become a common treatment method for CKD 2, 5, 6. The number of HD patients in Beijing region increased from 4406 to 11536 during a period of six years (2006 to 2012), which undoubtedly increased the burden of healthcare system. Although majority of the past studies has shown frailty to be a geriatric syndrome 1, 2, recent studies have suggested that patients with end-stage renal disease (ESRD) 7 or undergoing HD may have an increased risk for frailty at all ages 8. In addition, an American study has shown frailty to be a strong predictor of mortality and hospitalization in HD patients irrespective of age 9. However, there are very few studies investigating frailty as a survival predictor in Chinese population. Frailty in HD patients might be related to diverse factors such as impaired renal function 10, low body mass index (BMI), anemia, hypoalbuminemia, limitation in activities of daily livings and ambulation associated with a high risk of frailty in HD patients 11. Furthermore, several modifiable and preventable factors in frail HD individuals have been identified such as serum testosterone 12, state of acute illness and inflammation 13 etc. Hence, we could assume that frailty in HD patients could be reduced by targeting modifiable risk factor. National Kidney Foundation-Kidney Disease Outcomes Quality Initiative (NKF-KDOQI) 14 suggests that interdialytic weight gain should not exceed 4.8-5.7% of dry weight, we speculate more volume overload which is the risk factor of frailty for HD patients may develop a series of cardiovascular complications that lead to death.

This study aimed to investigate the prevalence and associated factors of frailty in Chinese HD patients, as well as the relationship between frailty and two-year survival.

## Materials and Methods

### Study participants

This prospective study enrolled patients admitted to the dialysis center of Xuanwu Hospital, Capital Medical University from October to December 2017 and followed up for two years. The data source for mortality was from our hemodialysis center database. All participants had a minimum of 2 years follow up and only one subject censored from the study because of kidney transplantation.

Subjects were assessed at the enrollment for physical frailty and divided into frail, pre-frail and non-frail groups. The inclusive criteria were that patients must undergo HD at least 3 months before recruitment, older than 18 years old and received hemodialysis three times per week during the follow-up period. The exclusive criteria were that patients with a history of hospitalization in recent 3 months on account of acute infection, acute heart failure, acute cerebral vascular disease, recent diagnosis of cancer, fracture or other severe diseases; or patients with a diagnosis of dementia and refusal to participate in this study.

### HD procedure

Patients underwent HD 3 times per week and 4 hours each time using the Fresenius 4008s machines with polysulfone membrane dialyzer and bicarbonate dialysate. Patients' vascular access included the following three types: autologous arteriovenous fistula, arteriovenous graft and tunnel-cuffed catheter. During HD, blood flow was set on 250-300ml/min, dialysate flow was set on 500ml/min. The hemodialysis adequacy means fractional urea clearance or single-pool Kt/V (spKt/V) was estimated using the formula derived by Daugirdas.

This study was approved by the institutional review board (IRB number file # 201112) of Xuanwu Hospital, Capital Medical University, Beijing, China. All enrolled participants signed the informed consents.

### Clinical features

Demographic data of each participant was collected at the enrollment including sex, age, education, marital status and dialysis vintage. Information on pre-existing conditions including diabetes mellitus (DM), hypertension (HT), coronary artery disease (CAD) and cerebrovascular disease (CVD) was assessed. Body mass index (BMI) was calculated using the measured height and dry body weight using the standard definition, i.e., BMI=weight (kg)/height^2^ (m^2^). Patients' blood pressure was measured within 10 minutes before the beginning and upon the completion of HD. Ultrafiltration volume and Kt/V was recorded.

Ultrafiltration volume is the amount of dehydrating water per hemodialysis. Dry weight is the body weight of a hemodialysis patient who expects to develop hypotension or shock with any further fluid removal. We checked patients' ultrafiltration volume, then divided by their dry weight and the ratio was recorded.

### Laboratory values

Laboratory data for blood included potassium (K), sodium (Na), calcium (Ca), phosphorus (P), CO2-CP, creatinine (Cr), urea, uric acid (UA), albumin (Alb), total cholesterol (TCH), triglyceride (TG), low-density lipoprotein (LDL), high-density lipoprotein (HDL), white blood cell (WBC), red blood cell (RBC) and platelet (PLT), hemoglobin (Hb). Fasting blood sample was taken before undergoing HD.

### Assessment of frailty phenotype

Frailty was assessed using the frailty phenotype by Fried and colleagues 1, as described elsewhere 13 and the criteria is based on Wu's study of frailty in Chinese population 15. In brief, 1. Weight loss: ≥5 kilograms in the past year; 2. Weakness (low grip strength): Female: ≤15.0kg (BMI ≤20.0kg/m^2^), ≤17.5kg (BMI 20.0-22.1kg/m^2^), ≤20.0kg (BMI>24.8kg/m^2^); Male: ≤25.2kg (BMI ≤20.6kg/m^2^), ≤28.5kg (BMI 20.6-23.2kg/m^2^), ≤30.0kg (BMI 23.2-25.9kg/m^2^), ≤30.0kg (BMI ≥25.9kg/m^2^); 3. Slowness (low gait speed over a level surface of 2.5 meter): Female: ≤0.36m/s (Height ≤151cm), ≤0.43m/s (Height >151cm); Male: ≤0.45m/s (Height ≤163cm), ≤0.48m/s (Height>163cm); 4. Exhaustion: Participants were asked “How often in the last week did you feel this way?” a) “I could not get going”, b) “I felt everything I did was an effort”. A score of 0 was given for a response of “rarely/none of the time; less than 1 day”, score of 1 was given for a response of “some or a little of the time; 1 to2 day”, score of 2 was given for a response of “a moderate amount of time; 3 to 4 days”, and score of 3 was given for a response of “most of the time”. Participants were considered to have exhaustion if they had a total score ≥4; 5. Inactivity: A self-report of < 10minutes of continuous walk during a usual week.

Subjects were classified as non-frail or robust (score = 0), pre-frail (score of 1-2), and frail (score ≥ 3) 1, 13.

### Statistical analysis

Variables were expressed as mean ± standard deviation, case numbers or percentages. Differences between groups (non-frail, pre-frail and frail groups) were analyzed using ANOVA and chi-square test. Spearman correlation analysis was used to assess the correlation between the frailty score and each parameter. Multivariate logistic regression analysis was used to identify the factors associated with frailty scores on HD patients and the variables that were statistically significant in univariate analysis (p<0.05) were included in multivariate logistic regression.

ROC curve was plotted with the frailty score as variable and death as the classification variable used to evaluate the predictive value of frailty score to two-year mortality. Kaplan-Meier was plotted by using the cutoff value obtained by ROC curve to evaluate survival rates in different frailty status. Survival analysis was conducted using Cox regression analysis. ROC curve analysis was performed by using MedCalc statistical software version 17.7 (MedCalc Software bvba, Ostend, Belgium; 2017). Statistical analysis was performed by using SPSS software (IBM SPSS statistics version 22). Statistical significance was set at *P* < 0.05.

## Results

### Participants and baseline frailty status

General demographics are presented in **Table 1**. 208 patients out of total 227 subjects were enrolled. Among the 208 patients, 113 were males (54.3%) and 95 were females (45.7%) with an average age of 60.5±12.7 years (age 18 to 83 years). One subject withdrew from the study because of a kidney transplant procedure. Dialysis vintage was 6-276 months with a median of 82.00 (69.00, 142.50) months.

### Prevalence of frailty and associated factors at baseline

**Figure 1:**
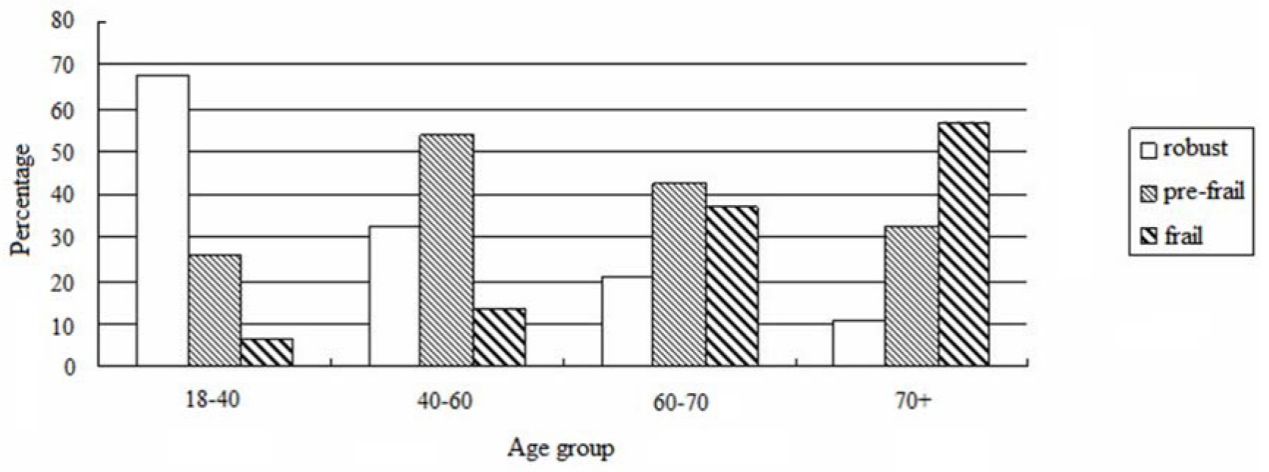
Prevalence of frailty according to age groups.

The prevalence of non-frail, pre-frail and frail subjects were 28.7%, 45.9%, and 25.4%, respectively. Frailty increased with age as per our analysis based on the four age groups (age 18-40:4.3%, age 40-60:10.5%, age 60-70:34.9%, age>70: 57.1%, *F* = 36.775, *P* < 0.001, **Figure 1**). In addition, frailty was associated with DM, CAD, CVD, decreased level of hemoglobin, uric acid, and albumin, and increased level of phosphorus (*P* < 0.05, **Table 1**). Although the ultrafiltration volume was not different among the 3 groups, frailty was significantly associated with an increased ratio of ultrafiltration volume to dry body weight (*P* < 0.05) (**Table 1**).

### Risk factors associated with frailty

Correlation analysis (**Table 2**) showed age, history of DM, CAD and CVD, blood phosphorus level and ratio of ultrafiltration volume to dry weight to be positively correlated with the frailty scores. Meanwhile, hemoglobin, albumin, uric acid and DBP after HD were negatively correlated with frailty. In the age-adjusted model, albumin, uric acid, DBP after HD and ratio of ultrafiltration volume to dry weight were also correlated with frailty.

In the multivariate logistic regression model, age (OR = 2.921, P < 0.001), ratio of ultrafiltration volume to dry weight (OR = 1.289, P = 0.035), uric acid (OR = 0.872, P = 0.032), hemoglobin (OR = 2.514, P = 0.026), albumin (OR = 2.863, P = 0.015) and diastolic pressure after hemodialysis (OR = 1.334, P = 0.011) remained associated with frailty (**Table 4**). The average value of the ratio of ultrafiltration volume to dry weight categorized into ≤ 0.039, 0.039-0.050, ≥0.050 three groups, the ratio of ultrafiltration volume to dry weight was found to be associated with an increased risk for frailty (OR = 1.725, P = 0.019) (**Table 4**). Moreover, patients who died during the follow up period had a higher ratio of ultrafiltration volume to dry weight than those who survived (**Table 3**).

### Association of frailty with mortality in HD patients

During the follow-up period, a total of 39 subjects died. The underlying causes of death included coronary artery disease (CAD, n=23), cerebrovascular disease (CVD, n=7), hyperkalemia (n=2), severe infection (n=2), malignant tumor (n=2) and others (n=3). Mortality was significantly associated with older age, higher frailty score, CAD comorbidity, increased ratio of ultrafiltration volume to dry weight and decreased level of hemoglobin (**Table 3**).

Multivariate cox regression analysis confirmed the continuous frailty scores (per 1 point) and non-frail (score=0), frail (score=3-5) to be independently associated with survival. Meanwhile, pre-frail (score=1-2) was proved to be irrelevant to survival (**Table 5**). Kaplan-Meier Survival curve has shown that survival time was shortened as the severity of frailty increased (**Figure 3A**) and there was still statistical significance after covariates adjustment (covariates include age, ratio of ultrafiltration volume to dry weight, Hb and CAD) (**Figure 3B**).

### Predictive accuracy of frailty in HD patients

ROC curve demonstrated that frailty (score ≥4) could predict two-year mortality. The Youden index is 0.5298. The sensitivity is 70% (95% CI: 45.7%-88.1%) and specificity is 83.67% (95% CI: 70.3%-92.7%) with an AUC of 0.819 (95% CI 0.708-0.902) (P <0.001).

**Figure 2:**
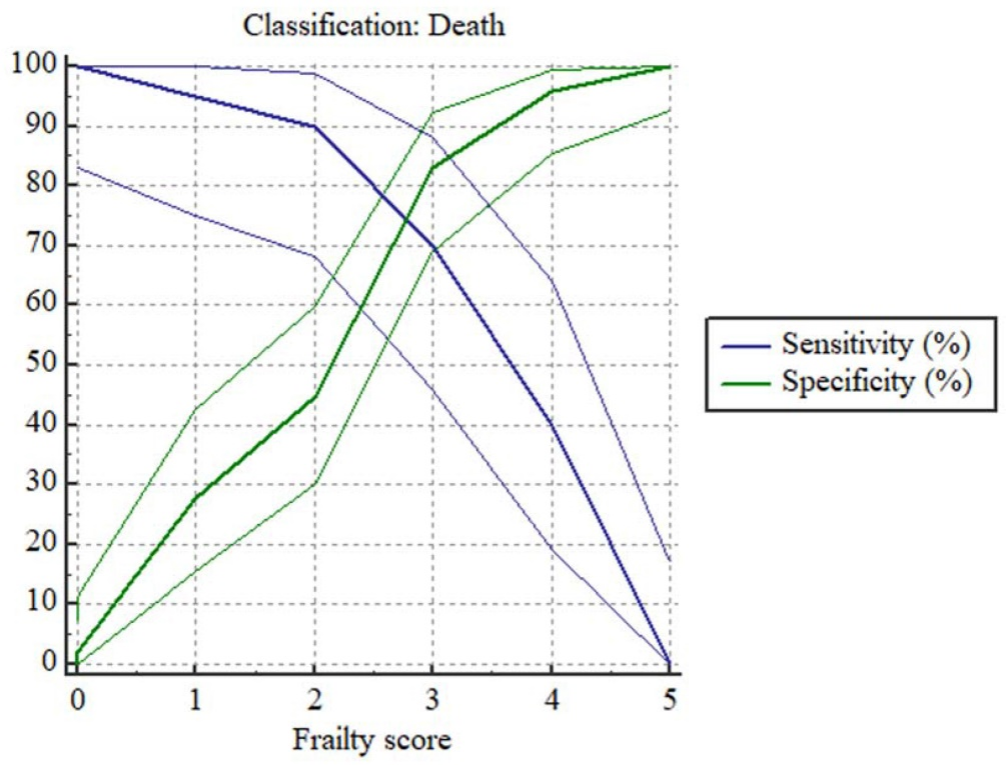
Plot versus criterion values based on frailty score in predicting two-year mortality of HD patients. Notes: Data of frailty score are presented as numbers and death is set as 1, survival is set as 0.

**Figure 3:**
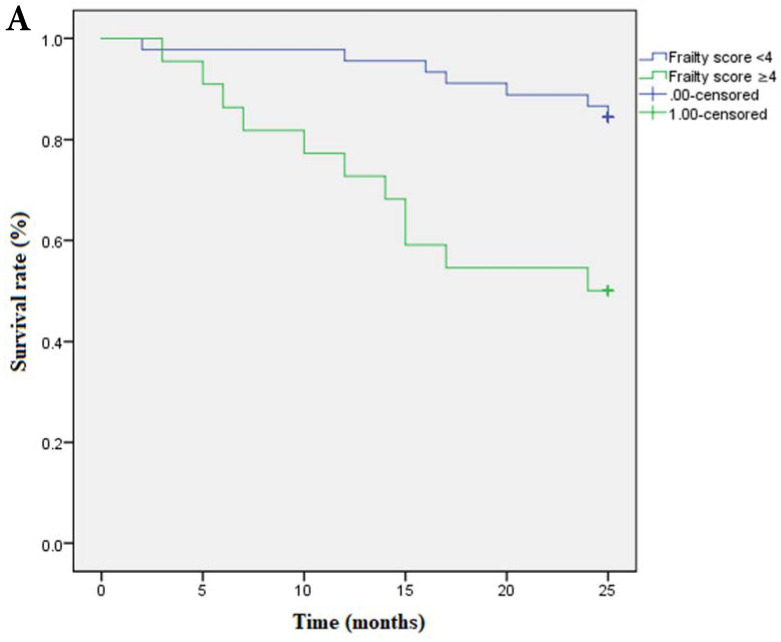

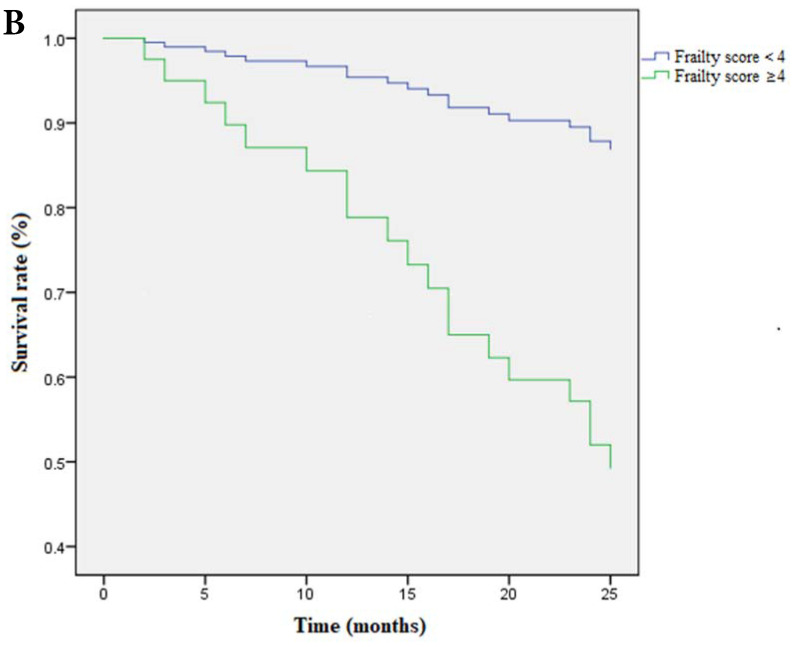
** A Kaplan-Meier curve based on frailty scores and mortality. Note:** Frailty score≥4 is set as 1, <4 is set as 0; death is set as 1, survival is set as 0. OR of frailty score≥4 is 1.680 (95%CI:15.025-21.612); OR of frailty score<4 is 0.675 ( 95%CI: 22.365-25.013). P=0.001. **B Survival curve based on frailty scores and mortality after adjusted for covariates. C**ovariates include age, ratio of ultrafiltration volume to dry weight, Hb and CAD. Data of frailty score are presented as numbers and death is set as 1, survival is set as 0. **Note:** Frailty score≥4 is set as 1, <4 is set as 0; death is set as 1, survival is set as 0. OR of frailty score≥4 is 1.673 (95%CI:16.109-21.633); OR of frailty score<4 is 0.690 (95%CI: 22.307-25.011). P=0.001.

We also evaluated the sensitivity and specificity of each frailty score in predicting all-cause mortality of HD patients with Plot versus criterion values (**Fig 2**) and the data were shown in **Table 6**.

## Discussion

Frailty has been acknowledged as a major risk factor for disability in older adults in China 16. The current study demonstrated a high prevalence of frailty among HD patients, which was a significant predictor for increased mortality risk in multivariable analysis. Although the diagnostic criterion of frailty is a score of ≥3, we found the optimal value for mortality prediction was a frailty score of ≥4. Age was the most important risk factor for both frailty and mortality in HD patients. A higher ratio of ultrafiltration volume to dry weight and lower level of blood hemoglobin increased the risk for both frailty and mortality. In addition, CAD was a major risk factor for mortality, while lower levels of albumin and uric acid, and decreased diastolic pressure after hemodialysis were independently associated with frailty.

Previous reports have shown the prevalence of frailty in HD patients to be approximately 14-81% 7, 10, 12, 17-20. In the current study, the prevalence of frailty was found to be lower than that of many European studies in HD patients 12, 17-20. However, similar findings were reported in Japanese HD patients 21. Such great variance could primarily be due to the varied measures used to assess frailty. In our study, as measured by the frailty phenotype the pre-frail and frail subjects accounted for 45.9% and 25.4% HD patients. In our study the prevalence of frailty was much higher in older patients although younger HD patients also had a higher rate of pre-frailty status which is consistent with previous studies 20, 22, 23. Moreover, it should be noted that the prevalence of frailty in community-dwelling Chinese older adults aged 60 years old or more was reported to be around 7.0% 24-26. While chronic comorbidities include CAD, CVD and DM initially associated with increased risk for frailty in our HD patients, but logistic regression analysis excluded them to be independent risk factors.

It is well recognized that cardiovascular complications and infection are the two leading causes of death in Chinese HD patients and our study came to a similar conclusion. An interesting finding of the current prospective study is that severity of frailty (frailty score ≥4) could predict all-cause mortality in two-year follow-up with a sensitivity of 70% and specificity of 83.67%, which to our knowledge has not been reported previously in the Chinese HD patients. In recent years, several studies have confirmed the relationship between frailty and mortality in HD patients 9, 20, 27-29. The predictive validity of frailty on survival has been verified in patients after surgery 30, 31. Previous studies 32-34 have explored the prognostic factors for short-term all-cause mortality in elderly patients with HD include lower serum albumin, lower BMI, higher level of C-reactive protein (CRP), suboptimal initiation of HD, malnutrition and frailty. But whether they are independent risk factors in HD patients has not been conclusively determined.

In the current study, survival analysis and Cox regression analysis both confirmed the correlation and predictive effect of frailty on mortality. Besides CVD which was not independently associated with frailty, CAD and both increased ratio of ultrafiltration volume to dry weight and lower level of blood hemoglobin were contributed to frailty and mortality. Cox regression analysis showed that only frailty score was an independent risk factor for mortality. Previous studies on the relationship between frailty and outcome have been limited to the elderly population. The current study demonstrated the independent predictive value of frailty for mortality in HD patients.

Apart from age, factors such as decreased levels of serum albumin, hemoglobin, uric acid, and DBP after HD were associated with frailty, and we have for the first time demonstrated a correlation between the ratio of ultrafiltration volume to dry weight and frailty and mortality. In general, ultrafiltration volume often represents volume load and is determined according to interdialytic weight gain clinically. Moderate ultrafiltration can stabilize blood pressure and reduce cardiovascular mortality. In the same way, massive dehydration during HD can lead to intradialytic and even post-dialytic hypotension, which may induce hypoperfusion and myocardial ischemia due to myocardial contractility 35. Patients with heart failure are known to have a very high prevalence (nearly 50%) of frailty 36.

In our two-year follow-up, among the 39 patients who died, death occurred mainly in patients with higher frailty scores and a higher ratio of ultrafiltration volume to dry weight. Considering CAD was the leading cause of death in our study, the above evidence supports our finding on the correlation of ratio of ultrafiltration volume to dry weight and frailty and suggest that long-term control of volume overload may be one of the effective measures to overcome frailty and even mortality in HD patients. According to KDOQI guideline 14, in clinical practice, we often empirically think that the ratio of ultrafiltration volume to dry weight not more than 4.8-5.7% can benefit patients. Our study found that the ratio of less than 3.9% is more conductive to reduce the prevalence of frailty. These may suggest that more stringent controlling of volume overload can improve the quality of life and prognosis of HD patients.

Our study also validated the relationship between nutrition and frailty. As we know, hypouricemia, hypoalbuminemia and anemia are all also markers of malnutrition. Evidence on the association of anemia and frailty in HD patients is scarce. A recent study in Arab HD patients has showed anemia as a predictor of frailty 20. Nonetheless, anemia is one of the most common complications in HD patients and higher risks of transfusion have been found in anemic patients compared to others 37. Previous investigations have shown subjects with lower hemoglobin level to have a higher risk of frailty, which was much prominent in older adults 38, 39. Hence, we could conclude that a decrease in hemoglobin level may contribute to frailty even in HD patients.

Serum albumin is a widely used marker of nutrition and found to be associated with frailty 40 and poor outcomes in various conditions including HD 13, 41. Our findings are similar to that of previous studies 18, 41, showing hypoalbuminemia to be related to frailty in HD patients.

Based on these above findings, HD patients should strictly limit fluid intake and strengthen nutritional support to reduce the incidence of frailty and improve survival rates. In addition, few studies have investigated the relationship between uric acid and frailty in HD patients previously. Although higher concentrations of uric acid have been found to increase the risk of frailty in older adults 42, we found HD patients with a lower level of uric acid to be associated with frailty and hypouricemia is common in HD patients with poor nutritional status 43.

The primary limitation of our study is that it was a single-centered study with a relatively small sample size. Thus, future multi-centered, large scale prospective studies are needed to confirm our findings which could be generalizable to the Chinese population. In addition, we did not take in account of psychological factors such as anxiety and depression which are known to impact frailty in patients with chronic diseases 44, 45. However, it could be speculated that the concomitant presence of psychological disorders in frail HD patients certainly will not lead to a good prognosis. Nevertheless, our study has highlighted several key points regarding frailty in HD patients in Chinese population.

In conclusion, our result confirms severity of frailty was a significant predictor of mortality in HD patients. Old age, malnutrition, and low blood pressure are associated with frailty. Interdialytic weight gain induced excessive ultrafiltration volume is an important risk factor. Our findings provide valuable references for clinicians to improve the quality of life and prognosis of HD patients. In addition, there is a need to strengthen geriatrics 46 and knowledge of age related syndromes such as frailty in all aspects of medicines to meet the care needs of vulnerable older population including in HD.

## Figures and Tables

**Table 1:** Comparison of demographics at enrollment based on frailty status

Items	Robust (n=60)	Pre-frail (n=95)	Frail (n=53)	*P* value
Male, n (%)	33 (55.0%)	52 (54.7%)	28 (52.8%)	0.982
Age (years)	51.7±11.6	59.5±10.1	70.9±8.5	<0.001*
Dialysis vintage (months)	69.00 (50.00, 115.00)	76.50 (61.00, 139.50)	92.50 (73.00, 157.25)	0.354
Kt/V	1.7±0.6	1.6±0.6	1.6±0.5	0.882
BMI (kg/m^2^)	21.35±4.55	20.89±3.65	20.24±4.13	0.557
DM	20 (33.3%)	33 (34.7%)	30 (56.6%)	0.045*
HT	44 (73.3%)	71 (74.7%)	39 (73.6%)	0.638
CVD	13 (21.7%)	23 (24.2%)	23 (54.7%)	0.023*
CAD	17 (28.3%)	45 (47.4%)	40(75.5%)	0.015*
K (mmol/l)	4.38±0.56	4.59±0.52	4.55±0.49	0.761
Na (mmol/l)	139.7±3.22	141.2±2.87	141.5±3.09	0.553
Ca (mmol/l)	2.27±0.21	2.25±0.17	2.15±0.22	0.336
P (mmol/l)	1.39±0.59	1.55±1.03	1.82±1.05	0.037*
Cr (μmol/l)	864.4±55.79	803.55±59.30	751.8±61.25	0.629
Alb (g/l)	45.94±6.76	39.21±8.29	35.33±6.14	0.025*
UA (μmol/l)	501±9.76	452±13.26	395±11.91	0.016*
TG (mmol/l)	2.25±0.29	2.19±0.35	2.27±0.31	0.465
TCH (mmol/l)	4.23±0.72	4.20±0.95	4.19±1.01	0.562
LDL (mmol/l)	2.15±0.49	2.07±0.81	2.19±0.66	0.592
HDL (mmol/l)	1.52±0.48	1.49±0.45	1.55±0.62	0.883
WBC (×10^9^)	5.98±1.22	6.15±1.43	6.39±1.30	0.714
Hb (g/l)	117.3±10.21	102.3±11.36	98.3±5.44	0.001*
PLT (×10^12^)	179±10.36	181±11.24	176±11.02	0.469
Before HD				
SBP (mmHg)	142.39±18.51	144.01±15.79	141.59±17.97	0.315
DBP (mmHg)	80.77±13.25	79.54±12.81	74.01±13.16	0.093
MAP (mmHg)	98.82±12.43	99.24±13.08	96.16±11.52	0.781
After HD				
SBP (mmHg)	128.30±12.55	125.56±13.19	121.68±13.45	0.225
DBP (mmHg)	73.84±10.57	71.25±12.41	69.94±10.31	0.292
MAP (mmHg)	93.07±9.59	90.95±11.56	88.26±11.39	0.149
Ultrafiltration volume (L)	4.5±2.1	4.7±2.6	4.5±2.5	0.628
Ultrafiltration volume/dry weight	0.038±0.019	0.043±0.027	0.069±0.022	0.036*

**Abbreviations**: BMI, body mass index; DM, diabetes mellitus; HT, hypertension; CAD, coronary artery disease; CVD, cerebral vascular disease; Alb, albumin; UA, urine acid; TG, triglyceride; TCH, total cholesterol; LDL, low density lipoprotein; HDL, high density lipoprotein; WBC, white blood cell; Hb, hemoglobin; PLT, platelet; HD, hemodialysis; SBP, systolic blood pressure; DBP, diastolic blood pressure; MAP, mean artery pressure. * P< 0.05 **Note:** Fried phenotype frailty classification: Robust or non-frail: frailty score=0; pre-frail: frailty score=1 or 2; frail: frailty score≥3.

**Table 2:** Spearman correlation analysis with frailty scores.

Variables	Unadjusted		Age-adjusted	
	*r* value	*P* value	*r* value	*P* value
Age (years)	0.392	<0.001*	-	-
DM	0.297	0.043*	0.281	0.099
CVD	0.533	0.034*	0.107	0.283
CAD	0.493	0.039*	0.137	0.255
Hb (g/l)	-0.606	<0.01*	-0.312	0.063
Albumin (g/l)	-0.405	<0.01*	-0.456	<0.01*
P (mmol/l)	0.240	<0.01*	0.292	0.074
Uric acid (μmol/l)	-0.730	<0.01*	-0.368	0.026*
DBP after HD (mmHg)	-0.482	0.020*	-0.424	0.015*
ultrafiltration volume/ dry weight	0.440	<0.01*	0.457	<0.01*

DM, CVD, CAD: no=0, yes=1 **Abbreviations**: BMI, body mass index; DM, diabetes mellitus; CVD, cerebral vascular disease; CAD, coronary artery disease; DBP, diastolic blood pressure; HD, hemodialysis. * P< 0.05

**Table 3:** Comparison of demographics at enrollment based on survival status during follow up

Items	Survival (n=168)	Death (n=39)	*P* value
Male, n (%)	92.4(55.0%)	20(51.3%)	0.838
Age (years)	58.5±9.2	67.3±7.7	0.019*
Dialysis vintage (months)	70.50(50.25, 106.50)	89.00(70.25, 157.25)	0.296
Kt/V	1.6±0.6	1.6±0.7	0.998
BMI (kg/m^2^)	22.29±3.52	21.03±4.16	0.772
DM	67(39.6%)	16(41.0%)	0.754
HT	123(73.2%)	31(79.5%)	0.327
CVD	48(28.6%)	11(28.2%)	0.934
CAD	72(42.9%)	30(76.9%)	<0.001*
K (mmol/l)	4.42±0.52	4.97±0.55	0.675
Na (mmol/l)	140.2±3.01	141.5±2.59	0.520
Ca (mmol/l)	2.21±0.22	2.19±0.23	0.501
P (mmol/l)	1.51±0.75	1.70±0.55	0.179
Cr (μmol/l)	834.67±55.59	856.05±62.14	0.388
Alb (g/l)	40.59±8.03	38.01±6.53	0.159
UA (μmol/l)	443±11.05	412±10.26	0.172
TG (mmol/l)	2.23±0.33	2.19±0.31	0.397
TCH (mmol/l)	4.21±0.55	4.15±0.95	0.504
LDL (mmol/l)	2.15±0.74	2.12±0.32	0.603
HDL (mmol/l)	1.52±0.44	1.49±0.57	0.825
WBC (×10^9^)	6.15±1.38	6.06±1.45	0.682
Hb (g/l)	115.3±11.02	99.6±7.03	0.014*
PLT (×10^12^)	180±11.44	181±9.73	0.598
Before HD			
SBP (mmHg)	140.51±15.42	142.22±17.04	0.353
DBP (mmHg)	79.52±13.09	72.89±12.41	0.084
MAP (mmHg)	97.33±13.15	95.56±12.04	0.809
After HD			
SBP (mmHg)	125.97±13.32	120.53±13.15	0.258
DBP (mmHg)	71.76±12.01	69.26±12.35	0.272
MAP (mmHg)	91.88±11.53	88.25±12.41	0.225
Ultrafiltration volume (L)	4.3±2.9	4.6±2.6	0.359
Ultrafiltration volume/dry weight	0.035±0.021	0.072±0.026	0.019*
Frailty score	2.12±1.82	4.07±1.27	<0.001*
Robust, n(%)	58(34.5%)	1(2.6%)	<0.001*
Pre-frail, n(%)	84(50%)	11(28.2%)	<0.001*
Frail, n(%)	26(15.5%) #&	27(69.2%) #&	<0.001*

**Abbreviations**: BMI, body mass index; DM, diabetes mellitus; HT, hypertension; CAD, coronary artery disease; CVD, cerebral vascular disease; Alb, albumin; UA, urine acid; TG, triglyceride; TCH, total cholesterol; LDL, low density lipoprotein; HDL, high density lipoprotein; WBC, white blood cell; Hb, hemoglobin; PLT, platelet; HD, hemodialysis; SBP, systolic blood pressure; DBP, diastolic blood pressure; MAP, mean artery pressure. * P< **0.05**; # **P**< **0.05** compared to non-frail; &** P**< **0.05** compared to pre-frail group

**Table 4:** Multivariable logistic regression analysis of factors associated with frailty.

Variables	OR	95% CI	*P*
Age (years)	2.921	1.553-2.384	<0.001*
DM	1.008	0.997-1.006	0.382
CVD	0.855	0.312-2.320	0.753
CAD	0.911	0.520-1.583	0.737
Hb (g/l)	2.514	1.132-4.297	0.026*
Albumin (g/l)	2.863	1.695-5.102	0.015*
P (mmol/l)	0.965	0.659-1.582	0.401
Uric acid (umol/l)	0.872	0.772-0.995	0.032*
DBP after HD (mmHg)	1.334	1.073-1.688	0.011*
ultrafiltration volume/dry weight (no groups)	1.289	1.087-1.531	0.035*
ultrafiltration volume/dry weight (3 groups)	1.725	1.101-1.425	0.019*

**Abbreviations:** BMI, body mass index; DM, diabetes mellitus; CVD, cerebral vascular disease; CAD, coronary artery disease; Hb, hemoglobin; DBP, diastolic blood pressure; HD, hemodialysis; OR, odds ratio; 95%CI, 95% confidential interval. * P< 0.05 **Note:** grouping of ultrafiltration volume /dry weight: ≤ 0.039, 0.039-0.050, ≥0.050. 0.039 is the average ratio of ultrafiltration volume to dry weight in non-frail group and 0.05 is a well-known clinical safety standard.

**Table 5:** Multivariable cox regression analysis of associated factors with mortality

Variables	OR	95%CI	*P* value
Model 1			
Age (years)	1.017	0.968-1.067	0.511
Frailty score	1.869	1.118-3.123	0.017*
Ultrafiltration volume/dry weight	5.340	0.015-15.231	0.827
Hb (g/l)	0.990	0.955-1.026	0.578
CAD	4.014	0.514-33.072	0.182
Model 2			
Age(years)	1.015	0.971-1.061	0.169
Non-frail	ref	ref	0.014*
Pre-frail	0.000	0.000-	0.984
Frail	0.199	0.068-0.585	0.003*
Ultrafiltration volume /dry weight	1.080	0.025-4.833	0.769
Hb(g/l)	0.996	0.936-1.002	0.936
CAD	0.472	0.000-7.458	0.159

**Abbreviations**: Hb, hemoglobin; CAD, coronary artery disease; OR, odds ratio; 95%CI, 95% confidential interval. **Notes**: Non-frail (score 0)is used as reference and is set as 0, Pre-frail (score 1-2)is set as 1, Frail (score≥3) is set as 2. Death is set as 1, survival is set as 0 * P<0.05

**Table 6:** Sensitivity and Specificity of each frailty score in predicting all-cause mortality of HD patients

Criterion	sensitivity	95%CI	specificity	95%CI	Youden index
0	100.00	83.2-100.0	0.00	0.0-7.5	0
1	100.00	83.2-100.0	2.13	0.05-11.3	0.0213
2	95.00	75.1-99.9	27.66	15.6-42.6	0.2266
3	90.00	68.3-98.8	44.68	30.2-59.9	0.3468
4	70.00	45.7-88.1	82.98	69.2-92.4	0.5298
5	40.00	19.1-69.3	95.74	85.5-99.5	0.3574

**Abbreviations**: 95%CI, 95%confidential interval. **Notes**: AUC of ROC curve is 0.819 (95% CI 0.708-0.902) (P < 0.001). Value of Youden index = value of sensitivity+ value of specificity-100.
